# Healthcare Assistants: distributional losses as a consequence of NHS modernisation?

**DOI:** 10.1111/ntwe.12053

**Published:** 2015-12-09

**Authors:** Ian Clark, Amanda Thompson

**Keywords:** contingent control regimes, exploitation, health care, labour process, NHS modernisation, work intensification, workplace resistance

## Abstract

This paper examines the labour process of Healthcare Assistants (HCAs) at a National Health Service (NHS) hospital trust (TUH) in the context of the NHS modernisation agenda. It determines whether application of the modernisation agenda is formalised at TUH and considers how HCAs are affected. The paper is based upon 60 interviews with HCAs, structured questionnaires completed by all interview respondents, observation of HCAs and interviews with non‐clinical managers. The findings show that elements of the modernisation agenda are informally implemented at TUH to the detriment of HCAs. HCAs experience distributional losses in the form of intensification as nurses deflect duties to HCAs and insulate themselves from adverse effects. HCAs resist, using selective absence when pressures mount. They ameliorate losses by re‐internalising their work as a job with caring elements not a genuine caring role. They rationalise their altered behaviour towards patients by blaming the regime's treatment of them as a subordinated group.

## Introduction

The term ‘NHS modernisation’ provides a narrative that describes a strategic approach to workforce management introduced by the UK Labour government in 1997 (Bach *et al*., 2007; 2008). This included a 10‐year NHS investment plan that tied higher spending to an explicit modernisation and reform agenda focused on role redesign and associated labour flexibility. Institutions such as the NHS Modernization Agency prompted initiatives such as the NHS Human Resources Plan (Department of Health, 2002), the NHS Plan (Department of Health, 2000) and ‘Agenda for Change’ (Department of Health, 1999), which implemented a service wide job evaluation scheme designed to modernise work practices and associated pay grading to place the patient at the centre of healthcare (McBride *et al*., 2005). A key aspect of modernisation was the further diffusion of the HCA's role under the rubric of skill mix that aims to re‐balance the deployment of professionally qualified and regulated staff with unregulated unqualified staff. The diffusion of HCAs continued uninterrupted through both Labour Government and Conservative Liberal Coalition Governments (1997–2015). The recently published Cavendish Report (2013) and The Francis Report (2013) have articulated the strengths and weaknesses of the HCA role in the aftermath of the failures in nursing care, such as those widely reported at the Mid‐Staffordshire Hospital.

The NHS modernization agenda creates a new internal division of labour that deploys graduate nurses away from the delivery of care towards administrative, technical and supervisory roles. Aspects of nursing work are now routinised and standardised and in effect delegated to HCAs (Bosley and Dale, 2008: 119). NHS modernisation and associated skill mix strategies redefine some regulated healthcare professionals as managers, for example, nurse ward managers. These roles divide professionals into competing groups to focus on leadership, empowerment and delegated support for HCAs who are increasingly responsible for direct patient care. As Bolton (2005: 6–7) observes, these changes have the effect of ‘making‐up’ managers in the NHS under a ‘care as a quality experience for patients as customers theme’ (Department of Health, 2000). Furthermore, it is argued that this ‘making up’ translates to the informal implementation of substantive losses for HCAs where under the auspices of ‘care as a quality experience’ they experience a form of work intensification (Bolton, 2004; Bosley and Dale, 2008).

The policy aim of NHS modernisation is to move the frontier of workplace control decisively in favour of hospital management (Harrison, 2002). The programme and its effects, however, are contested and controversial. In evaluating the purpose and consequences of the programme for registered nurses and HCAs, many contributions to the literature focus on efforts by registered nurses to sustain occupational closure over HCAs as a subordinate group (Daykin and Clarke, 2000). Other contributions examine the effects of efforts towards occupational closure by registered nurses on the role and profile of HCAs as modernisation develops (Bach *et al*., 2012). These contributions thus major on what appears to be a tense relationship between registered nurses and HCAs within the NHS as a result of modernisation. What remains less clear however is how do HCAs experience modernisation in their daily work?

An additional way in which we utilise the term NHS modernisation relates to its use as a strategy to carry through neoliberalism in the political economy of the state. Best defined as the opposite of embedded liberalism that was centred on a political commitment to full employment, an inclusive welfare state, workers' rights and decent wages enforced by collective bargaining, neoliberalism is focused on the market (Harvey, 2007: 11–12; Mason, 2015: 4–5). Application of the market mechanism to embedded liberalism projects rights for workers and decent wages for clearly defined jobs as standing in the way of capitalist revival. We use labour process theory to examine how abstract changes in capitalist political economy, such as NHS modernisation are experienced by HCAs by interrogating the effects of the changes upon the work systems and practices of both managers and workers in the employment relationship. It follows from this that the technical implications of these changes are central concerns of labour process theory: they relate to the conditions in which work is performed, who controls the work of HCAs and the skills these workers possess and how they are paid.

Modernisation seeks to implement a Fordist labour process because it aims to centralise management control over HCAs by delineating job instructions and job labels into hands‐on routine and advanced tasks and hands‐off relational care. This approach aims to provide certainty in the delivery of routine caring and advanced caring and denies that incumbent approaches centred on clinical freedom could provide such certainty. In this context, as the NHS modernisation programme has developed, occupational groups with greater positional power such as doctors and registered nurses have been able to protect their labour process at the expense of HCAs lower down the caring hierarchy. It is appropriate therefore to explore the nature of the distributional losses HCAs experience as a consequence of NHS workplace modernisation and how they seek to protect themselves.

The next section of this article outlines the focus for the research and reviews the extant literature that informs the study. We then present the research methodology, followed by our findings and discussion, before drawing conclusions.

## Research focus

In this article we examine the technical aspects of the HCA role. Reliance on new technology associated with patient care record systems and the delivery of related routine and advanced care tasks has become a central bureaucratic strategy of control over the labour process in health care, particularly in hospitals. Hence our research questions weave together how technology, the work of HCAs and the potential for intensification of the HCA employment relationship can explore the lived experiences of HCAs under modernisation. We stress that HCAs, as the weakest occupational group, stand least chance of being able to deflect intensification of the labour process and other negative worker consequences flowing from the modernisation agenda. In addition we are interested in the way in which the labour process has been managed and manipulated and the extent to which outcomes are formally or informally orchestrated.

By utilising elements of Bélanger and Edwards' (2013) approach to front‐line service workers, the theoretically informed empirical part of this study captures the presence of substantive but informal distributional losses suffered by HCAs as work intensification. Bélanger and Edwards (2013) reinforce the imperative of focusing on the distinctive features of the employment relationship that remain significant within and beyond service work such as call centre work and nursing broadly defined. They go on to argue that what is often presented as a post‐structural field of inquiry and research (the worker‐customer interaction) remains part of the employment relationship not an adjunct to it. That is, pressures to be customer focused and the measurement of employee performance in terms of customer service metrics are enacted and structured within the employment relationship not independently of it in a customer–worker relationship. Indeed, this focus and associated metrics flow from the technical aspects of the HCA role that the NHS Modernisation Agency highlighted as a redesigned and extended role ‘to attract and retain an effective workforce’ (NHS, Department of Health 2004: 97–111). So within the NHS where the performance of health care as service work emphasises the centrality of the quality of patient care, the healthcare practitioner–patient relationship should be not be regarded as independent to the HCA employment relationship, rather it should be seen as an integral part of the work HCAs undertake.

The framework employed in this paper thus recognises the importance of concentrating on the central structural status of the employment relationship to reveal how the HCA labour process is characterised and how modernisation affects this characterisation beyond and independently of a central focus on patients as clients or customers. Firstly, the employment relationship in the workplace is indeterminate and therefore gives rise to the imperative of management control (Edwards, 1986: 5). A key point, however, is that Fordism only works when formally institutionalised in the workplace (Clark, 2001). So a first research question focuses on the extent to which local implementation of the NHS modernisation strategy is formally institutionalised.

Secondly, structured antagonism remains a key feature of the employment relationship within and beyond the imperative for front‐line service workers such as HCAs to be patient focused. So a second research question focuses on the technical aspects of the HCA role where a subordinate group is affected by modernisation and seeks to identify the processes that drive the outcomes of modernisation for HCAs. Thirdly, this article builds on a particular conclusion of Bélanger and Edwards (2013) to argue that while front‐line service workers such as HCAs retain intrinsic job satisfaction in the face of work intensification, they may do so in a more instrumental and informally self‐defined manner. So a third research question centres on the extent to which HCA experience of work intensification locally stimulates forms of resistance which the Fordist aspirations of modernisation are less able to accommodate.

## HCAs, modernisation and distributional losses

In a six‐country study of HCAs Appelbaum and Schmitt (2009) found that, irrespective of location in either market‐led or coordinated economies, efforts to reduce costs saw a greater reliance on the use of HCAs in the delivery of primary health care in hospitals. The study found that these efforts drove down wages, degraded nursing care and expanded the use of low wage HCA jobs universally but found more inclusive employment relations systems reduced the severity of the low‐road trajectory. In the UK, Bach *et al*. (2008) identify variability of experience in the detail of HCAs roles beyond the modernisation agenda to highlight competing models and technologies of nursing care that reflect different assumptions about registered and non‐registered nursing roles.

Other contributors label these changes as flexibility, which stems in part from registered nurses focusing their work time away from hands‐on nursing in favour of the management of medication compliance, associated paperwork and scheduling (McBride *et al*., 2005; Hancock and Campbell, 2006). More specifically, Hyde *et al*. (2005: 704) examine how role redesign in the health service degrades nursing care wherein HCAs substitute for registered nurses. This theme is also highlighted by Nancarrow and Borthwick (2005), who report that unqualified, non‐graduate HCAs now undertake tasks previously performed by registered nurses. This evidence base also demonstrates that the central work activity of many registered nurses is focused beyond direct nursing care and in turn results in components of both routine and advanced nursing care becoming routinised and delegated to a subordinate group below registered nurses.

In the absence of a clearly defined workplace role for HCAs, more strategic efforts at job redesign across the health service are impeded. Kessler *et al*. (2010; 2013) report that changes in the role of registered nurses has created a legitimate space for HCAs yet the absence of a clearly defined job role for HCAs in the modernisation agenda creates the potential for overlapping HCA typologies ranging from ‘bedside technician’, ancillary, citizen, all‐rounder to expert. Any strategic underpinning in the utilisation of HCAs varies across trusts leading to a variety of outcomes for stakeholders including HCAs themselves (Kessler *et al*., 2012).

It is the failure to ground centrally formulated strategy on workplace utilisation of HCAs in policies and procedures that in turn leads to significant variations in the role of HCAs and the work they undertake, training for this work and resourcing of the role. A lack of central strategy to inform implementation of modernisation also gives rise to the possibility that work intensification and resistance to it is likely to be variable, in part because of the informal ways local managers impose or negotiate these losses on HCAs.

## Research methodology

### Research setting

Recruited as unqualified support staff, the term HCA describes staff who may work towards National Vocational Qualifications in health care at level two, which is equivalent to General Certificate of Secondary Education level or level three which is equivalent to advanced level study or ‘A levels’. However, study for this vocational qualification is not compulsory or a requirement of the job. HCAs provide the bulk of ‘hands‐on’ care in hospitals and make up a third of all caring staff in hospitals. In 2012, there were between 106 500 to 270 000 HCAs in the UK providing support to doctors and nurses. There are, however, over 60 job titles that may or may not cover HCAs hence the wide span of possible numbers (Health and Social Care Information Centre, 2012; Cavendish, 2013: 6, 15); it is also notable that there is no nationwide job description for HCAs and no national register for HCAs as there is for nursing staff. HCAs can undertake a wide range of nursing and direct patient care duties under the delegated task‐by‐task supervision of registered nurses. Despite their unqualified status, the HCA labour process is divided between routine tasks and advanced tasks. Routine tasks cover making beds, helping patients with bathing and eating, monitoring and recording glucose levels, taking patient temperature and pulse and so forth. Advanced tasks include catheterisation, cannulisation, complex dressings, machine monitoring and responses, administering injections and taking blood. In addition to these roles, HCAs perform relational caring such as reassuring patients and their relatives.

In terms of pay, while originally excluded from the collective bargaining framework, HCAs are employed on bands 1–3 in the Agenda for Change framework where the band above (band four) represents an assistant practitioner grade. Nationally, 56 per cent of HCAs are paid on band two. Thirty of the HCAs we interviewed are positioned on band three (£16 110–£19 077) of the pay scale, and the other 30 are on band two (£14 153–£14,846). In comparison, the minimum starting salary for registered nurses in 2012–2013 was £21 176 placing the minimum starting salary at the bottom of band 5 (Royal College of Nursing, 2012). Therefore, in 2012, the minimum starting salary for a registered nurse was £2099 higher than the maximum HCA salary, excluding overtime payments. Table 1 compares key HCA statistics in this study to the national picture detailed in Cavendish (2013: 15) and a recent study of four trusts for the National Institute for Health Research, (Kessler *et al*., 2010). The development and diffusion of the HCA role poses several challenges to hospital managers, ward managers, regulators, patients and HCAs themselves, not least the safeguarding of patient safety and issues related to the execution, monitoring and measurement of job performance.

**Table 1:** Key statistics for Healthcare Assistants

	Cavendish Review National Numbers (2013)	TUH (2012)	Kessler et al. (2010) 4 trust study
Average age	45	38	42.6
Gender	84% female	96% female	91% female^a^ (95–81)
Average years in post	4.1 in England	6.1 (TUH is in England)	9^a^ (all four trusts are in England)
Pay	56% on band 2	50% on band 2 and 50% on band 3	83%^a^ on band 2 (90–80)

a Averages calculated from tables 7 and 11, pages 57 and 65, highs and lows in brackets.

### Data collection and analysis

The fieldwork interviews were undertaken throughout July 2012. Teaching and University Hospital (TUH) is a large teaching and university‐affiliated hospital trust and employs 8,000 people. The hospital has 1,000 inpatient beds, 30 operating theatres and a 100 + bed critical care unit and is recognised as one of Europe's leading hospitals with an international reputation for care quality, informatics and clinical training and development. TUH was one of the first hospitals to secure NHS Foundation Trust status in 2004 and currently treats 90 000 inpatients a year. The hospital has an in‐house call bank for HCAs and in addition to this often uses agency staff. In‐house bank HCAs either work very flexibly at short notice as a form of zero hours spot contracting or permanent HCAs do bank work as a form of overtime.

The method of data capture was fourfold; firstly, 60 semi‐structured interviews were conducted with HCAs. The interviews were framed around the three research questions: local implementation of modernisation, intra‐group structured antagonism and resistance to local implementation of modernisation. The research questions emerged from a wider literature review, which revealed that these themes had hitherto not been reported in detail from a labour process perspective or with a focus on HCAs. Responses were coded in sub‐categories across the three questions. Interviews averaged 75 minutes, the shortest was 45 minutes and the longest was 2 hours. A second method of data capture was secured by a structured questionnaire completed by all interviewees. The topics included in the questionnaire covered the following issues: length of service, age, gender; band and length of service in band; reasons for absence over the past three years, related absence management and exposure to the Bradford formula; and relationships with nurses and ward managers. In addition to this, approaches to teamwork on wards and in outpatients departments; job satisfaction rankings and workload responsibilities in skill mix all featured in the questionnaire. The final part of the questionnaire asked questions on acceptable and unacceptable task delegation for band 2 and band 3 HCAs, requested examples of both forms of practice and questioned HCAs on the impact of absence on co‐workers.

A third source of data capture was provided by workplace observation of nurses and HCAs over a two‐week period in order to elicit an enhanced appreciation of workplace relations and the lived experiences of healthcare workers at TUH. More specifically, observation provided the possibility to control for some of the more dramatic claims made by a few HCAs in interviews and in questionnaire responses. Nurses too are subject to work intensification under modernisation so it is not possible to assume that the perspective of all HCAs is necessarily accurate on every issue. Lastly, five interviews with senior non‐clinical managers in TUH's delivery team provided further context to the local issues faced by the hospital and the role definition of HCAs.

## Empirical findings

We report our empirical findings around the themes outlined in our research focus to highlight the local implementation of modernisation at TUH and the extent to which this demonstrated workplace de‐skilling and work intensification. We then go on to report patterns of resistance to work intensification.

### Local implementation of modernisation at TUH

Forty‐five questionnaire responses stated that modernisation made the HCA role ‘just a job’ in two senses, one that highlighted limited pay and reward opportunities, and a second that stated that limited learning and development opportunities built into modernisation strategies further exacerbated these problems. In interviews, HCAs argued that ‘you can't really get off grade 3’ (HCA 41) irrespective of their length of experience (nine HCAs had more than 10 years of experience in the job and four of these had over 20 years of experience). Eighty‐three per cent of structured questionnaire responses reported poor pay and few training opportunities. Hence, for many HCAs, modernisation further intensifies their role and gradually encouraged them to see their work as so routinised that it became ‘just a job’.

At TUH, HCAs experience modernisation as a highly localised boundary issue. In their daily roles HCAs report intensification of routine and advanced tasks whereas at a more macro level they view the Trust's use of bank and agency staff to solve patient delays and cope with the absence of regular staff as part of a locally contingent regime to manage the delivery of care. A focus on these issues highlights the argument made by many HCAs that managers and nurses do not step in to alleviate HCA's workloads when there are staff shortages. In interview findings and questionnaire transcripts, it proved difficult to find responses that cited or referred to specific TUH policies that defined the boundary between the work of registered nurses and work that was delegated to HCAs. The absence of documentary evidence on this and the absence of information to the contrary in separate interviews with non‐clinical managers in the delivery team led to the conclusion that the job roles of registered nurses and HCAs had not been formally defined or redesigned to any nationally defined standard. The interviews with the delivery team did reveal the following:

In the absence of national guidelines we have recently revised job descriptions for bands two and three locally. This revision included guidelines designed to remove doubt on what could be legitimately delegated to band two and band three HCAs. (Head of delivery, TUH)

Interview responses from HCAs regularly included the claim that ‘They (nurses) just don't do that’, with reference to activities that registered nurses apparently will not undertake such as escorting patients to the toilet, changing soiled bed linen and helping patients with eating and drinking. Forty responses stated that registered nurses have redefined themselves as ‘schedulers’ and managers that provide distance from the HCA role and disassociate themselves from ‘dirty work’ and other routine but degraded tasks that might be classified as below their status. In interviews, HCAs went so far as to suggest that registered nurses were not perturbed by delays to patient care caused by absence or capacity issues, typical among these was: ‘delays create another “non‐nursing” job for them to do which can be massaged and legitimised in performance management figures’ (HCA 14).

### Internal sub‐contracting; de‐skilling and work intensification

#### De‐skilling

In interviews with HCAs and in questionnaire transcripts, respondents defined themselves as cheaper workplace substitutes for registered nurses and accepted their roles in the performance of a wide range of work spanning routine to advanced tasks. Many HCAs did not however accept that routine tasks such as rotation of patients, help with eating and drinking, washing and bed sore control were de‐skilled merely because HCAs performed them rather than registered nurses. Questionnaire responses to a definition of de‐skilling and the extent of de‐skilling in HCA job roles saw two‐thirds of respondents state that this was an inappropriate term to use. For example, while advanced tasks were added to work rosters undertaken by HCAs, respondents labelled this not as de‐skilling but rather work intensification stating that advanced tasks such as catheter maintenance could not be de‐skilled in terms of job content. We followed this point up in interviews where the following was a typical response:

‘nurses won't *because they can't* perform some tasks’ … ‘this is part of the problem of how we experience modernization and skill‐mixing’. … ‘they are more skilled than us but don't want to do it’ (HCA, 23)

This HCA appeared to argue that registered nurses, that is, those theoretically more skilled than HCAs are unable or unwilling to perform these tasks but should be able to do so because of their skill. A recurring survey response was ‘nurses don't help’, a response recorded in 45 questionnaires. So HCAs as individuals in a work group experience modernisation and skill mix strategies at TUH as work intensification prompted by staff shortages, related workplace stress and associated pressures from managers and registered nurses over the frequency of task‐by‐task routine and advanced task delegation. This is evidenced in the following comments:

‘… in out‐patients slots are missed and they don't get seen or have to wait a long time so you feel you have to get them there but you might not’ (It's not always like this but can be) (HCA, 23)

… You spend a lot of time walking around pushing patients; this gets to be heavy work. Sometimes schedules are so tight you are in effect running pushing a patient or running to get a patient from their ward to where they need to be. (HCA, 41)

Registered nurses were not interviewed or surveyed so we are unable to comment on this occupational group as authoritatively as we do on HCAs. Our workplace observations did however confirm that some registered nurses and nurse ward managers do get involved, whereas others would not get involved in the delivery of tasks they labelled as ‘HCA work’ for two reasons. Firstly, as a form of resistance to protect their distributive gains via workload re‐allocation and secondly because there often appeared no point so‐doing if staff shortages are such that they will lead to delays and/or cancellations regardless. More significantly, our observation revealed that the time registered nurses spend inducting, training and supervising HCAs helps them define the labour process of a subordinate group.

#### Work intensification

As a work group, HCAs experience intensification of routine tasks at TUH resulting from faster patient turnover and greater use of day surgery year on year without a corresponding increase in registered nurse numbers (see also Bolton, 2004: 324). More significantly, intensive repetition of routine tasks appeared to serve as a form of workplace ‘learning by doing’. Consequently, this paved the way for further deployment and diffusion of work away from registered nurses to HCAs and in turn to either work intensification for HCAs or delays for patients when HCAs were absent.

Fifty per cent of survey respondents stated that having to manage staff shortages and the consequent intensification of routine tasks and related workload stress resulting from these shortages was a reason for their own selective use of absence from the workplace. Here selective use of absence by HCAs was an attempt to mount resistance to efforts by registered nurses to delegate more advanced tasks to them. Hence, as an individual response HCAs who prefer to operate in the traditional HCA role of ancillary or bedside technician selectively chose absence when they knew there was likely to be a ‘shift‐shortage’ of HCAs (this phrase was cited by numerous HCAs). By choosing absence, these HCAs avoided further work intensification of routine and advanced tasks but clearly contributed to the intensification of work and stress experienced by their fellow HCAs on shift. Thus, while modernisation might be a process, in terms of the Kessler *et al*. (2010; 2013) typology referred to earlier, some HCAs seek to maintain themselves as traditional HCAs as a form of resistance to work intensification in the local regime at TUH. So while at TUH, workers may populate different typologies of HCA the further they move away from routine tasks towards advanced tasks, the more the labour process is likely to be intensified. Similarly, the closer HCAs stay to traditionally defined ancillary nursing and routine tasks, the more they are likely to exhibit resistance to the diffusion of modernisation in the workplace.

HCAs taking this course of action admitted that their absence and restricted job performance intensified the work of their colleagues but defended their position on the basis that it happened to all of them now and then. It follows from this that absence creates ‘the script’ (HCA, 34) ‘we are short staffed’, whereas the shortage of staff necessitates what Bolton (2005) terms managers ‘making it up’. Within this scenario, managing patient expectations involves ‘getting it across early’ (HCA 45) that a delay or a cancellation is routine.

Thirty survey respondents stated that delays in patient care and cancellations could be avoided if managers and nurses stepped in to help out. Most of these respondents refused to accept the finding from workplace observation cited above that nurses and wards managers themselves were subject to work intensification. Similarly, many respondents refused to accept that delays may persist in some cases even if nurses step in to help HCAs. It was evident that some HCAs held deeply entrenched positions on this issue that several of them maintained in the face of evidence that established that not all registered nurses refused to undertake ancillary nursing tasks.

### Resistance to work intensification

At TUH, HCAs experience modernisation as alienation from more traditional ancillary nursing roles such as bedside care and related work roles often termed ‘patient involvement’. Alienation such as this did, however, lead HCAs to accommodate a wider, if variable exploitation of their lowly workplace status. A ‘sticky floor’ confines HCAs due to limited opportunities and career development. HCAs expressed frustration at having to act like registered nurses and the fact that in order to get the job done, particularly the advanced tasks, bedside time with patients had to be compromised. This behaviour demonstrates a display of resistance by HCAs and a grudging acceptance that as a result of modernisation they were being called on to adjust their understanding of their own role from one of a ‘caring job’ to a job with some caring elements. Typical interview responses reported ‘the compulsion’ to act like registered nurses while they had to accept that they had ‘no chance’ (HCA 5) of becoming one. HCA 5 was previously highly motivated but admitted that she had recently begun to pull back from this position as a form of resistance to work intensification and to display frustration at the lack of promotion opportunities. Furthermore, the ‘dead‐ end’ (HCA 7) status of the job, which was frequently remarked upon, gave further licence to more exploitation and efforts to secure greater work intensification. One HCA added that TUH had in the past (before all‐graduate nursing status) put up to 20 of its best HCAs into nursing programmes annually but now this route had virtually closed down (HCA 5). Many referred to the closure of this route of progression and HCA 7 stated that ‘job satisfaction at work would be better if the vocational career pathway still existed and the potential for pay band improvement’.

These frustrations encouraged HCAs to cope by disengaging from caring at work—the ‘just a job’ response as opposed to ‘this is a caring job’. In effect, the demands of the role compelled HCAs to create their own distributive gains, which they sought out individually in the workplace, for example, by ‘putting the “block‐hole” on chat’ that is not slowing down or taking the time to chat with patients (HCA 21) or making patients wait for delivery of routine tasks where the imperative of advanced tasks dictated a priority. In essence, while HCAs still sought intrinsic job satisfaction and wanted to do a good job, the only way they could cope and effectively buffer themselves from the stresses of work intensification was to become increasingly instrumental in their work and unitise patients as occupants of bays or bed numbers;

I simply didn't have the time or capacity to spend time getting to know patients as individuals. (HCA 33)

Three quarters of survey responses recorded job satisfaction as satisfactory or better and nine respondents recorded job satisfaction as less than satisfactory but not unsatisfactory with six respondents recording job satisfaction as unsatisfactory. It appears to be the case that HCAs have redefined what they mean by job satisfaction to highlight that they do a good job and enjoy the work but to dissociate the effects of their instrumental responses in terms of its effects on the quality of service provision. Therein potential delays, cancellations, and distributive gains and losses that HCAs self‐receive in the form of more instrumental intrinsic job satisfaction are achieved by squeezing time for hands‐off nursing care.

Workplace observation established that nurses spend considerable amounts of time on technical and clinical governance issues such as managing patient care in the process of throughput for admission and discharge. Workplace observation found these technical issues were not only time consuming but also expansive in the event of cancellations, delays and re‐scheduling, and create what Cooke (2006) describes as ‘dis‐empowering’ effects of empowerment, that is, audit and paper trail compliance. So we can at least infer that nurses too are subject to work intensification and protect themselves from this by demarcating routine and some advanced tasks as ‘HCA work’, rather than their own. Nurses are able to balance the losses they incur by the imposition of new tasks (previously undertaken by junior doctors) by shedding other tasks to HCAs. This protects nurses from work intensification and gives them a distributive gain (or at least a neutral effect) in the chain of distribution of tasks. Cooke (2006) sees this as part of a re‐configured disciplinary process in nursing where work allocation to HCAs as well as the deployment of HCAs is imposed on them as a form of quasi‐formal discipline. In contrast to registered nurses, HCAs do not have a comparable opportunity to protect themselves against distributive losses as they are at the bottom of the hierarchical chain without a subordinate occupational group to whom to further delegate tasks. This is illustrated by the following:

on many wards where I have worked nurses and the delivery people are like the railways they can always find a good defendable reason to explain why something hasn't been done. (HCA 10)

the way you are given things to do varies a lot from ward to ward sometime not by much but it does you can be dumped on to find yourself doing things. (HCA 55)

A third of our survey responses stated that, under modernisation, it was a struggle to cope with the work intensification effects of managed labour shortages. A quarter of respondents recorded that they felt stressed by the managed labour shortages that stemmed from localised efforts by nurses to delegate part of their advanced tasks to HCAs to prevent their own work intensification. Examples cited included being bullied into undertaking advanced tasks, operating as carriers of news about cancellations or missed slots and having to run around the hospital to get things done.

## Discussion and conclusion—HCAs modernisation, informality and resistance

Bélanger and Edwards (2013) argue that front‐line service workers remain embedded within an indeterminate employment relationship where employers and their agents, in this case non‐clinical managers, nurse ward managers and registered nurses, continually seek control over subordinates, in this case HCAs. Efforts towards control structure workplace antagonism where control strategies (in this case NHS modernisation) result in work intensification for subordinate groups. This in turn stimulates resistance by employees. In turn, resistance and its effects demonstrate the indeterminate nature of management control in the workplace. The key point raised by Bélanger and Edwards, which has central relevance to this study, focuses on the manner in which front‐line service workers can retain job satisfaction. In this case, how HCAs feel satisfied with the level of patient satisfaction and service that they deliver. What this study adds to this framework is a detailed illustration of what happens in a workplace if centralised strategies and related technologies for work are not effectively grounded in the application of formalised policies and procedures. In addition to this, the study also demonstrates how local contingency allows HCAs to reformulate their views of job performance by imposing some of the effects of work intensification they experience on patients whilst retaining job satisfaction and a realigned form of patient focus. Our application of Bélanger and Edwards demonstrates that relations with customers—in this case patients—do create frustration and dissatisfaction in the employment relationship. There are however challenges in this application. NHS modernisation places patients at its core but patients do not directly shape the development of modernisation or the HCA role. Therefore, it is necessary to locate the labour process of front‐line service workers such as HCAs in the political economy of neoliberalism pursued by the state. Here the focus on choice and consumer sovereignty—in this case the centrality of patients—reinforces the foundation of conflict as in this case the contribution of HCAs is not reflected in their conditions of employment.

The work intensification and labour process consequences for HCAs that flow from modernisation at TUH are significant because the local control regime centres on efforts to secure task‐by‐task delegation of routine and advanced tasks to HCAs by registered nurses. Workplace agency exercised by HCAs provides structure to modernisation and associated informal structures in equally contingent resistance strategies that are deployed in a highly localised manner. Resistance is particularly visible because at TUH, the contingent mode of control is responsible for re‐allocating distributive gains for nurses and imposing these on HCAs as work intensification.

This study demonstrates that it is important to evaluate the reality of HCA experiences under modernisation and its informal implementation in one hospital. As Cavendish (2013) and The Francis Report (2013) establish, the failures in nursing care at Mid Staffordshire hospital stemmed from weaknesses in the HCA role that resulted from a failure to effectively ground the NHS modernisation strategy in the workplace. At TUH, there is no clear structure imposed by management, nor is one enforced by trade unions, hence the contingent approach where control rests with local management is not exercised through a rational and explicit framework. Rather the vacuum is filled by nurses accruing authority to make wards operate as they see fit often via the application of changeable settlements about work distribution. These initiatives reinforced the indeterminate nature of management control under modernisation where ‘being short staffed’ and its effects appear to represent the substance of management control problems.

In terms of the first research question, within the local implementation of modernisation, the frontier of workplace control moved decisively in favour of management at TUH, but a locally contingent approach to HCA deployment was evident. NHS modernisation aimed to transform an established clinically focused bureaucracy into a locally focused management‐led bureaucracy tasked with diffusing and imposing centrally formulated strategies designed to move the frontier of job control towards management. Failure to ground key aspects of this strategy in national standards of job design for HCAs led alternatively to the re‐creation of professional boundaries and clinical hierarchies. This demonstrates indeterminate management control and is evident in the difference between the formulation of strategy and its operational diffusion and implementation in a systematic manner at the workplace. The absence of national standards for HCA job design means that regulation, such as that at TUH, becomes locally contingent and informal within the workplace, often on a ward‐by‐ward basis. Contingency appears to govern efforts towards work intensification and shape the relationship between HCAs, registered nurses and ward managers in respect of delegation of routine and advanced tasks.

On the second research question, the demands of modernisation have stimulated pressures for greater management control as described above, yet the frontier of control is incomplete due in the main to the absence of national structures to formally manage the HCA role and guide local contingencies. The local mode of control and associated distributive gains and losses are a form of internal subcontracting enforced by ward managers and registered nurses as work intensification for HCAs. This intensification is partly made possible by a technical division of HCA work into routine and advanced tasks. This division is technologically and systematically backed, but we feel that those who impose work intensification on HCAs rely rather less on this backing but alternatively use attrition and force of personality to enforce intensification.

There is clear evidence of structured antagonism between HCAs and TUH as their employer and ward managers and registered nurses as their managers. Firstly, in the informal and variable work intensification exercised over HCAs by registered nurses in routine and advanced task‐by‐task delegation, secondly in the distribution of gains and closures created by this mode of control. So while modernisation at TUH demonstrates a locally contingent mode of regulation and does structure antagonism between HCAs, registered nurses and ward managers local contingency illustrates the failure to implement a Fordist conception of bureaucratic control that the NHS modernisation strategy aimed to implement. As the findings show the local regime does not de‐skill work because this is not possible for many advanced and some routine tasks, rather it seeks to de‐grade work but does so in an inconsistent fashion.

On the third research question of resistance, at TUH, the local regime for delegation of both routine and advanced tasks saw delivery team managers accept local responses to this as an individual or highly sectional act of resistance to work intensification imposed by registered nurses. This agency legitimises resistance in a more instrumental approach to work and illustrates how local informal control regimes shape individual acts of resistance. So while hospital managers, ward managers and registered nurses each concentrate on what flexibility means to their own interests, these interests usurp bureaucratised policies for job redesign, associated workplace change and formalised decision‐making. This approach curtailed vocational routes into registered nurse status while defining the work of HCAs on a task‐by‐task basis but under the control and supervision of registered nurses. The aim of this was to maintain distributive gains at the expense of work intensification and further exploitation of HCAs.

In this regime, HCAs retained intrinsic job satisfaction by marginalising aspects of their labour process, particularly hands‐off patient care, and so effectively reduced their distributive losses by imposing loss on those they sought to help. Therein, HCAs, like registered nurses, ultimately seek to make up their own distributive gains and blame their reduced patient focus on the intensified and exploitative regime which is imposed on them informally. Our research at TUH found that HCAs were right to do so, and in terms of further research one issue that deeply frustrates HCAs is the manner in which their work is conflated with that of nurses and referred to as nursing. Therefore, future research could usefully examine the feasibility of the Cavendish (2013: 36) proposals for a certificate of fundamental care for HCAs, what this might mean for technical aspects in the delivery of routine and advanced tasks and any potential flowing from such certification for intensified forms of working. Similarly, if information is the currency of health care and health workers in the future, recording national standards by certification for HCAs may also facilitate application of NHS modernisation strategies that are more appropriate than local contingency that relies on highly localised patterns of work intensification.
